# High performance clean versus artifact dry electrode EEG data classification using Convolutional Neural Network transfer learning

**DOI:** 10.1016/j.cnp.2023.04.002

**Published:** 2023-04-25

**Authors:** M.N. van Stigt, E.A. Groenendijk, H.A. Marquering, J.M. Coutinho, W.V. Potters

**Affiliations:** a Amsterdam UMC location University of Amsterdam, Department of Clinical Neurophysiology, Meibergdreef 9, Amsterdam, the Netherlands; b Amsterdam UMC location University of Amsterdam, Department of Neurology, Meibergdreef 9, Amsterdam, the Netherlands; c Amsterdam UMC location University of Amsterdam, Department of Biomedical Engineering and Physics, Meibergdreef 9, Amsterdam, the Netherlands; d Amsterdam UMC location University of Amsterdam, Department of Radiology and Nuclear Medicine, Meibergdreef 9, Amsterdam, the Netherlands

**Keywords:** Electroencephalography, Dry electrodes, Artifact, Convolutional neural network, Transfer learning

## Abstract

**Objective:**

Convolutional Neural Networks (CNNs) are promising for artifact detection in electroencephalography (EEG) data, but require large amounts of data. Despite increasing use of dry electrodes for EEG data acquisition, dry electrode EEG datasets are sparse. We aim to develop an algorithm for *clean* versus *artifact* dry electrode EEG data classification using transfer learning.

**Methods:**

Dry electrode EEG data were acquired in 13 subjects while physiological and technical artifacts were induced. Data were per 2-second segment labeled as *clean* or *artifact* and split in an 80% train and 20% test set. With the train set, we fine-tuned a pre-trained CNN for *clean* versus *artifact* wet electrode EEG data classification using 3-fold cross validation. The three fine-tuned CNNs were combined in one final *clean* versus *artifact* classification algorithm, in which the majority vote was used for classification. We calculated accuracy, F1-score, precision, and recall of the pre-trained CNN and fine-tuned algorithm when applied to unseen test data.

**Results:**

The algorithm was trained on 0.40 million and tested on 0.17 million overlapping EEG segments. The pre-trained CNN had a test accuracy of 65.6%. The fine-tuned *clean* versus *artifact* classification algorithm had an improved test accuracy of 90.7%, F1-score of 90.2%, precision of 89.1% and recall of 91.2%.

**Conclusions:**

Despite a relatively small dry electrode EEG dataset, transfer learning enabled development of a high performing CNN-based algorithm for *clean* versus *artifact* classification.

**Significance:**

Development of CNNs for classification of dry electrode EEG data is challenging as dry electrode EEG datasets are sparse. Here, we show that transfer learning can be used to overcome this problem.

## Highlights

• We present an automated single channel dry electrode EEG classification algorithm.
• Our CNN-based algorithm accurately classifies *clean* vs. *artifact* dry electrode data.
• Transfer learning enables CNN training for small-scale dry electrode datasets.

## 1 Introduction

Visual interpretation of clinical electroencephalography (EEG) data is time-consuming. To speed up and improve EEG interpretation, multiple machine learning algorithms for interpretation of EEG data have been developed (Hosseini et al., 2021). An important step in data preprocessing, especially for feature-based algorithms, is the rejection and/or correction of artifact data. (Gasser et al., 1992, Liu et al., 2018). Multiple methods have been proposed for this purpose, ranging from filtering to independent component analysis and neural networks (Jiang et al., 2019, Kim and Keene, 2019). Advantages of neural networks over most traditional methods include high accuracy (Sun et al., 2020), low computation time (Yang et al., 2018), and the ability to learn the best features from raw EEG data and perceive spatiotemporal characteristics of the data. Neural networks proposed so far often focus on specific artifact types or require multiple input channels (Cisotto et al., 2020, Kim and Keene, 2019, Paissan et al., 2022, Sun et al., 2020, Yang et al., 2018). Moreover, most of these algorithms are based on traditional wet electrode EEG data. In recent years, dry electrode EEG systems have been introduced for clinical use, and these systems are rapidly improving (Fiedler et al., 2022, Fiedler et al., 2015, Vasconcelos et al., 2021). Their use may be preferred in settings where fast and easy application of EEG electrodes is required, e.g. acute epilepsy diagnosis or prehospital stroke triage (van Meenen et al., 2021). Dry electrode EEG data are similar to wet electrode EEG data, but have slightly higher power in the lower frequency bands (<6 Hz), and are more susceptible to artifacts (Chen et al., 2014, di Fronso et al., 2019, Fiedler et al., 2022, Fiedler et al., 2015). Convolution Neural Networks (CNNs) are promising for automatic artifact detection (Boudaya et al., 2022) but these require large amounts of data. Although the use of dry electrodes for clinical applications is on the rise, large dry electrode EEG datasets are sparse. Therefore, we developed a CNN-based algorithm for *clean* versus *artifact* classification of dry electrode EEG data by transfer learning an existing CNN for classification of wet electrode EEG data.

## 2 Methods

### 2.1 Dataset

We acquired dry electrode EEG data in 13 adult subjects, of whom 10 healthy volunteers and 3 patients who visited the outpatient clinic of the department of Clinical Neurophysiology of Amsterdam University Medical Centers for routine medical care. EEG data were acquired using a dry electrode EEG cap with 8 Ag/AgCl coated electrodes in positions FC3, FC4, CP3, CP4, FT7, FT8, TP7 and TP8 (Waveguard touch, Eemagine, Berlin, Germany; Appendix A, Figure A.1), and a compatible EEG amplifier (eego amplifier EE-411, Eemagine, Berlin, Germany) at a sample frequency of 500 Hz. During data acquisition, physiological and technical perturbations were simulated to induce artifacts. These artifacts included eye blinks, eye movements, jaw clenching, talking, frowning, head movement, electrode cable movement and high electrode–skin impedances for the reference, ground, and cap electrodes. Total duration of the EEG recordings was 149 min and the median (IQR) duration per session was 11 (5–15) minutes. Detailed recording protocols are included in Appendix A.

The study was approved by the ethical review board of Amsterdam University Medical Centers (METC 2018_175). All patients provided written informed consent.

### 2.2 Data preparation

EEG data were re-referenced to a 12-channel bipolar montage with six bipolar channels located at each hemisphere (Appendix A, Figure A.1C). Data were then bandpass filtered between 0.5 and 35 Hz using a 3rd order Butterworth filter because most clinically relevant information is included in this frequency range. Subsequently, data were downsampled to 100 Hz, and extreme non-physiological values were excluded by clipping data at −800 and + 800 µV.

EEG data were sample wise annotated as *clean*, *clean with a high frequency component*, or *artifact* by 3 to 4 trained reviewers. Each bipolar channel was annotated separately, and margins of maximal 0.5 s were included. As a measure of inter-rater agreement, we determined Fleiss’ kappa for the data annotated by 3 reviewers and for the data annotated by 4 reviewers and calculated the weighted average. Thereafter, data annotated as *clean with a high frequency component* were automatically labeled as *clean* or *artifact*. Data with a small high frequency component^1^, most likely caused by environmental noise or weak muscle activity, were labeled as *clean*. Data with a large high frequency component, most likely caused by strong muscle activity, were labeled as *artifact*. All binary labels (*clean* or *artifact*) were, per sample, transformed to a soft label by the dividing the number of assigned *clean* labels by the total number of assigned labels (i.e. the number of reviewers). EEG data were then, per bipolar channel, split into 2-second segments with an overlap of 1.9 s. All soft labels within a 2-second segment were averaged to one soft label per segment.

We split the annotated EEG sessions in an 80% train and 20% test set using the Scikit-learn toolbox (Pedregosa et al., 2011). Multiple sessions of one subject were always assigned to either the train or the test set.

### 2.3 Algorithm development

We used a 1-dimensional CNN trained for *clean* versus *artifact* classification based on an open source wet electrode EEG dataset (Artifact EEG Corpus v2.00 (Hamid et al., 2020); Temple University Hospital Philadelphia, Pennsylvania) as pre-trained CNN (Appendix A, Figure A.2) (van Stigt, 2020) The open source dataset contains 310 EEG recordings in which artifacts were annotated as muscle artifact, eye movement artifact, instrumental artifact (i.e. electrode pop, electrostatic or lead artifact), chewing, shivering, or a combination of these. The pre-trained CNN was designed to classify single-channel inputs. Using our dry electrode train dataset, consisting of 2-second single-channel segments, we fine-tuned the pre-trained CNN three times using stratified 3-fold cross validation. Each time, the last layer of the CNN was trained from scratch, and the model weights of all other layers were fine-tuned. The CNNs were trained using AdamW optimizer (Loshchilov and Hutter, 2019) by minimizing the binary cross-entropy training loss, and cosine annealing was used to schedule the learning rate. The initial learning rate was set to 0.001. Each of the CNNs was trained for a maximum of 100 epochs with a relatively large batch size of 256 to speed up the training. Once the validation loss no longer improved for 10 consecutive training epochs, the training was stopped early. The optimal classification threshold for each CNN was based on the validation data and defined as the maximum recall at a precision of ≥ 90%. Finally, the three CNNs were combined in a *clean* versus *artifact* classification algorithm in which the majority vote of the CNNs was used for the classification of the 2-second EEG segments as *clean* or *artifact* data.

### 2.4 Evaluation of algorithm performance

The pre-trained CNN, the three fine-tuned CNNs, and the *clean* versus *artifact* classification algorithm were applied to the unseen test data. True labels were derived from the soft labels per segment: a soft label ≥ 2/3 was converted to a true label of 1 (*clean*), and a soft label < 2/3 was converted to a true label of 0 (*artifact*). CNN and algorithm performance were evaluated by accuracy, F1-score, precision, and recall. Additionally, we report the specificity of the *clean* versus *artifact* classification algorithm.

## 3 Results

**Fig. 1:**
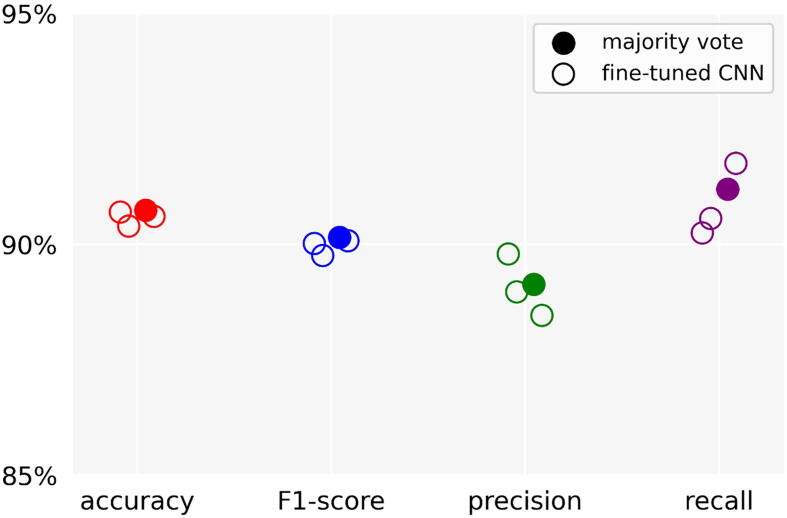
**Classification performance.** Accuracy, F1-score, precision and recall for prediction of clean electroencephalography data by the three fine-tuned Convolutional Neural Networks (CNNs) and their majority vote.

**Fig. 2:**
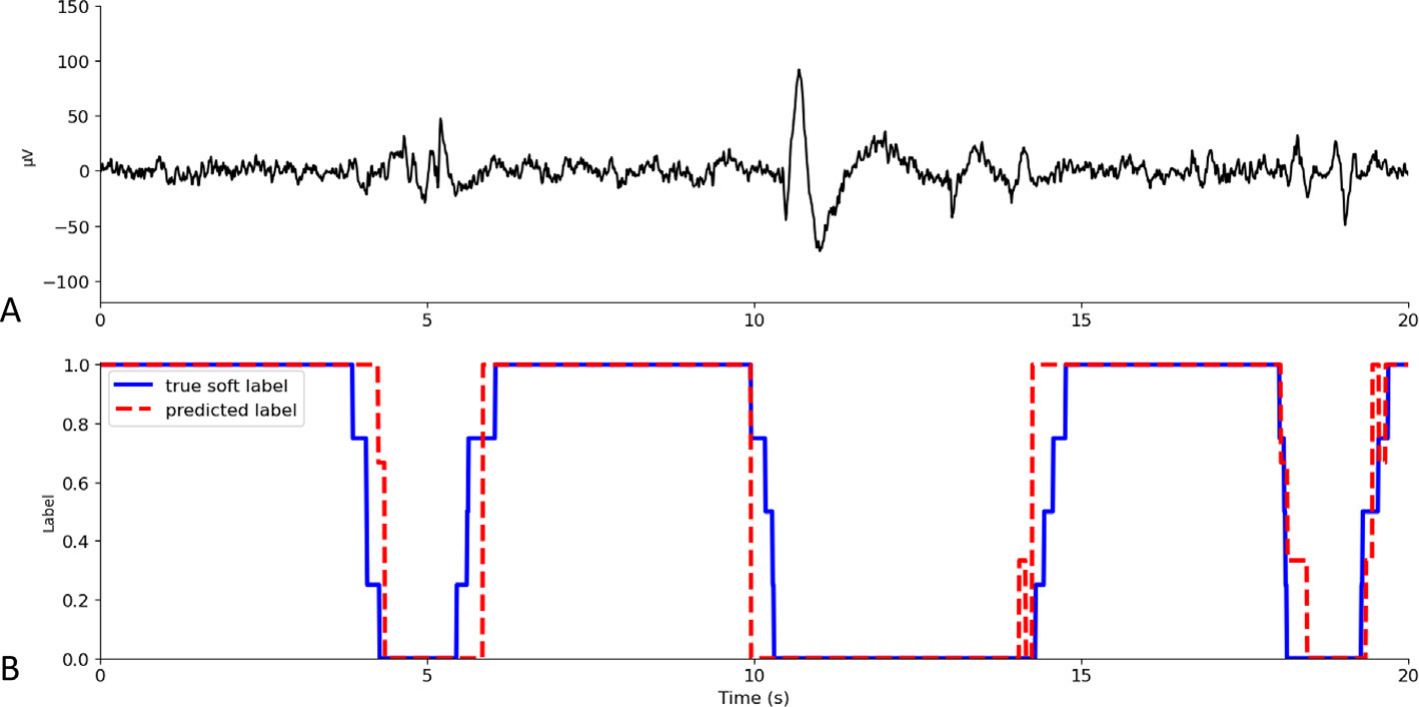
**Example electroencephalography (EEG) data, true soft labels, and predicted labels.** A. Example EEG data. B. True soft labels, i.e. the proportion of reviewers that labeled the data as clean (blue line), and predictions assigned by the *clean* versus *artifact* classification algorithm (red dashed line). (For interpretation of the references to colour in this figure legend, the reader is referred to the web version of this article.)

Fleiss’ kappa was 0.70, indicating substantial agreement between the reviewers whom annotated the data. The *clean* versus *artifact* classification algorithm was trained on 0.40 million 2-second segments (49% clean, 51% artifact) and tested on 0.17 million 2-second segments (46% clean, 54% artifact). On the test set, the pre-trained CNN had an accuracy of 65.6%, F1-score of 73.0%, precision of 57.5%, and recall of 99.9%. The *clean* versus *artifact* classification algorithm, using the majority vote of the three fine-tuned CNNs, had an accuracy of 90.7%, F1-score of 90.2%, precision of 89.1%, recall of 91.2% and specificity of 90.3% when applied to the test data (Fig. 1). Example EEG data with predictions assigned by the *clean* versus *artifact* classification algorithm and soft labels derived from the reviewer scores are shown in Fig. 2.

## 4 Discussion

We developed an automatic CNN-based *clean* versus *artifact* algorithm for single-channel dry electrode EEG data by fine-tuning a CNN that was trained for the same classification task in wet electrode EEG data. Fine-tuning increased the classification performance for dry electrode EEG data (accuracy pre-trained vs. fine-tuned algorithm: 65.6% vs. 90.7%).

Currently, artifacts in EEG data are often manually rejected, which is time-consuming. Our algorithm enables automatic and continuous analysis of large datasets where manual selection of clean data is not feasible. Other neural networks for detection of both physiological and technical artifacts in EEG data exist, but these have lower classification performance or are only applicable to multichannel EEG data (Cisotto et al., 2020, Kim and Keene, 2019, Peh et al., 2022). Existing single-channel neural networks have mostly been validated on semi-synthesized ocular and muscle artifacts only.(Paissan et al., 2022) Peh et al. developed a single- and multi-channel algorithm for artifact classification using a large clinical dataset with a wide variety of artifacts. The multi-channel algorithm outperformed the single-channel algorithm for the multiclass classification task. Binary classification (*artifact* versus *clean*) performance was only evaluated for the multi-channel algorithm and its sensitivity to artifact data was lower than observed in our study (Peh et al., 2022).

Single-channel algorithms, like ours, are preferred as these could be applied to high-density in-hospital EEG data, but also in settings where limited EEG channels are available. Moreover, our algorithm can be applied in settings where dry electrodes are used. Examples of the latter are the potential application of EEG in the ambulance (van Meenen et al., 2021) or homecare setting, where EEG data need to be acquired by relatively unexperienced users in limited time.

We expect that accurate clean data selection by our algorithm may aid in EEG interpretation and may improve the performance of an automated downstream EEG classification algorithm. The extent of improvement will most likely depend on the dataset, e.g. many or few artifacts, and the selected algorithm, e.g. feature-based or not, and should thus be studied for each application separately (Islam et al., 2021, Roy et al., 2019). Also, the accepted risk for artifact data predicted as *clean* should be considered per application.

Multiple factors contributed to the significant improvement in classification performance achieved by fine-tuning. First, dry electrode EEG data are prone to artifacts related to suboptimal electrode–skin contact which are less common in clinical wet electrode EEG data. Hence, the pre-trained algorithm was not optimally trained to detect these artifacts. Second, the background pattern of dry electrode EEG data differs slightly from that of wet electrode EEG data, with more power in the lower frequencies. Third, differences in the annotation of the pre-training and fine-tuning datasets led to variations in the assigned labels.

Our study has several limitations. First, our algorithm is based on data acquired at specific electrode positions, mostly central and temporal. The algorithm has thus not yet been validated for data containing artifacts that occur exclusively in the frontal or occipital region. Second, although we included a large variety of artifacts in our study, the algorithm has not yet been validated for cardiac, respiratory and sweat artifacts. Third, the algorithm only detects artifacts but does not correct them. Especially in case of sparse data, an artifact correction algorithm is preferred, provided that this algorithm has excellent performance, i.e. artifact data are (almost) completely removed and the background EEG data are reconstructed accurately. Otherwise, downstream (feature-based) classification algorithms could possibly be affected by the remaining artifact data. Single-channel neural networks for correction – rather than detection – of artifacts exist and have moderate to good performance (Mathe et al., 2021, Sun et al., 2020, Yang et al., 2018, Zhang et al., 2021). These algorithms are promising, but so far have only been validated on semi-synthesized data that contained ocular, muscle and/or electrocardiogram only.

## 5 Conclusions

Despite a relatively small dry electrode EEG dataset, transfer learning enabled development of a high performing CNN-based algorithm for *clean* versus *artifact* classification (accuracy: 90.7%; F1-score: 90.2%) in single-channel dry electrode EEG data.

## Funding

This work was supported by the Dutch Heart Foundation, Health ∼ Holland and by an unrestricted research grant from Medtronic. These funding sources had no role in the design of this study, its execution, analyses, interpretation of the data, and decision to submit results.

## Declaration of Competing Interest

The authors declare that they have no known competing financial interests or personal relationships that could have appeared to influence the work reported in this paper. HM is co-founder and minor shareholder of Nicolab and TrianecT. JC received related research support from the Dutch Heart Foundation and Medtronic and unrelated research support from Bayer and Boehringer (all fees were paid to his employer), and is co-founder and minor shareholder of TrianecT. WP is co-founder and minor shareholder of TrianecT. The other authors have no conflicts to report.

## Footnotes

1 Defined as maximum amplitude ≤50µV and the power in the lower frequency band (5–15 Hz) divided by the power in the higher frequency band (25–40 Hz) > 0.5, detected per 1-second bipolar EEG channel.
