# Supplementary material for: High performance clean versus artifact dry electrode EEG data classification using Convolutional Neural Network transfer learning
Source: Clin Neurophysiol Pract. 2023 Apr 25;8:88–91. doi: 10.1016/j.cnp.2023.04.002 (PMC10196906; doi:10.1016/j.cnp.2023.04.002)
Supplement: Supplementary data 1 [file mmc1.pdf]

APPENDIX A

High performance clean versus artifact dry electrode EEG data classification using Convolutional Neural Network transfer learning

M.N. van Stigt, MSc[1,2], E.A. Groenendijk, MSc[1,2], H.A. Marquering, PhD[3,4], J.M. Coutinho, MD, PhD[2], W.V. Potters, PhD[1,2]

1. Amsterdam UMC location University of Amsterdam, Department of Clinical Neurophysiology, Meibergdreef 9, Amsterdam, The Netherlands

2. Amsterdam UMC location University of Amsterdam, Department of Neurology, Meibergdreef 9, Amsterdam, The Netherlands

3. Amsterdam UMC location University of Amsterdam, Department of Biomedical Engineering and Physics, Meibergdreef 9, Amsterdam, The Netherlands

4. Amsterdam UMC location University of Amsterdam, Department of Radiology and Nuclear Medicine, Meibergdreef 9, Amsterdam, The Netherlands

Corresponding author:

M.N. van Stigt

Department of Clinical Neurophysiology

Amsterdam UMC location University of Amsterdam

Meibergdreef 9, Amsterdam, The Netherlands

Email: m.n.vanstigt@amsterdamumc.nl

RECORDING PROTOCOLS

Task	Duration (minutes)
**Protocol 1**	
Rest	1
Rest, eyes closed	1
Eye movements (horizontal, vertical)<br>Active eye blinking<br>Frowning<br>Chewing<br>Talking<br>Head movements (horizontal, vertical)	1
**Protocol 2**	
Rest	2
Rest, eyes closed	2
Active eye blinking	1
Eye movements (horizontal, vertical)	1
Jaw clenching (three different gradations)	2
Slightly disconnected electrode FC3	1
Slightly disconnected electrode FT7 (or CP3)	1
**Protocol 3**	
Rest	1
Rest, eyes closed	1
Reference electrode disconnected	1
Reference and ground electrode disconnected	1
Ground electrode disconnected	1
Active eye blinking	1
Jaw clenching	1
Talking	1
Head movement (horizontal, vertical)	1
Slightly disconnected electrode FC3 + intermitted jaw clenching	1.5
Slightly disconnected electrode CP3 + intermitted jaw clenching	1.5
Slightly disconnected electrode FC4 + intermitted jaw clenching	1.5
Slightly disconnected electrode CP4 + intermitted jaw clenching	1.5
**Protocol 4**	
Rest	1
Rest, eyes closed	3
Cap cable movement	0.5
Head movement (horizontal, vertical)	0.5
Active eye blinking	0.5
Continuous contraction of eyelid without opening eyes	0.5
Jaw clenching	0.5
Talking	0.5
Disconnect one cap electrode	2

Eyes open, unless otherwise specified.

A. B. C.

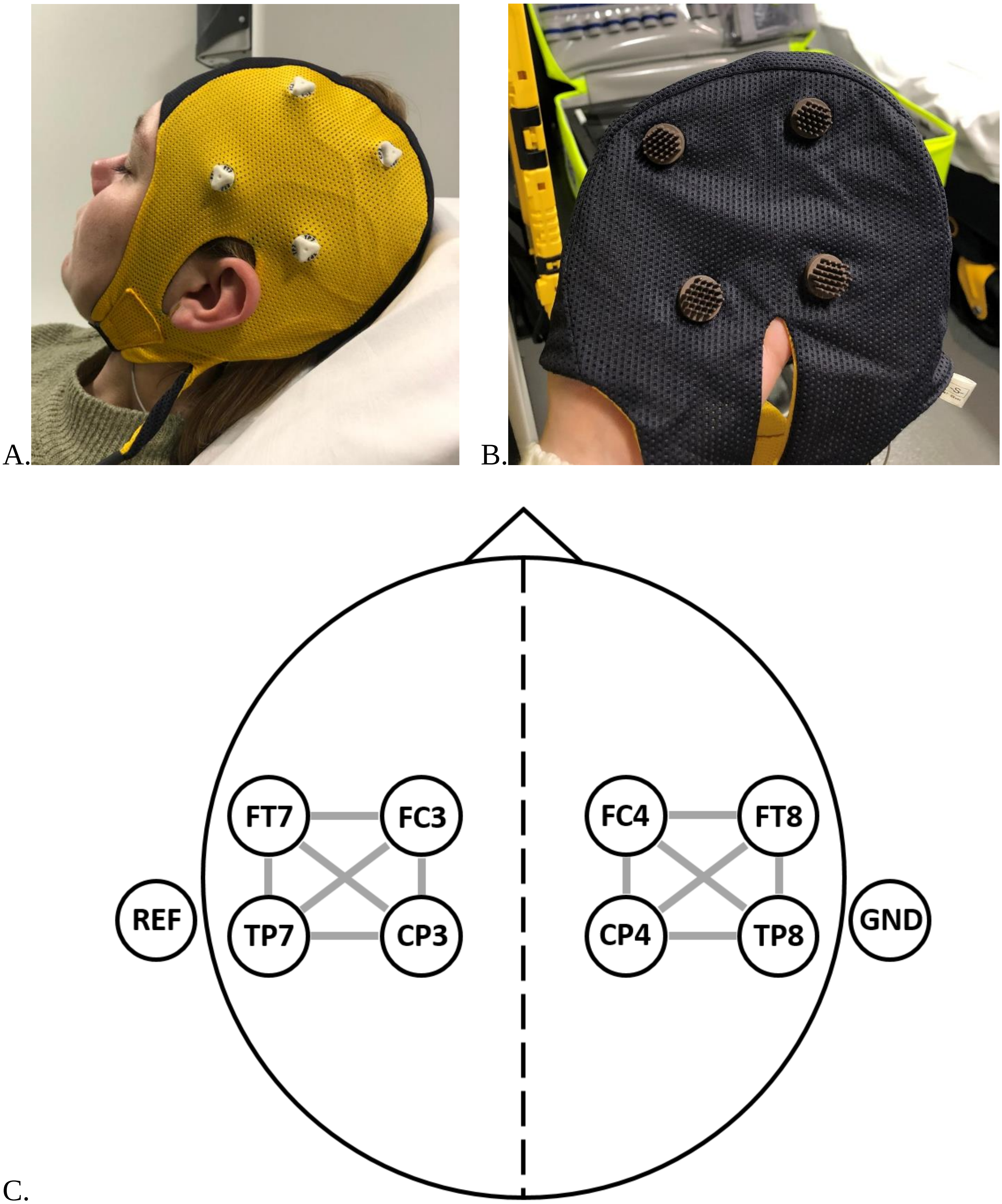

Figure A.1. Dry electroencephalography cap (Waveguard touch, Eemagine, Berlin, Germany). A. Outside of the cap. B. Inside of the cap with the multipin Ag/AgCl coated dry electrodes. C. Electrode positions with the twelve bipolar channel derivations visualized in light grey.

Conv1d (#filters 8; kernel size 9)
BatchNorm1d
max_pool1d
relu
Conv1d (#filters 8; kernel size 9)
BatchNorm1d
max_pool1d
relu
Conv1d (#filters 8; kernel size 9)
BatchNorm1d
relu
Dropout (probability=0.39)
Linear
Linear

Figure A.2. 1-dimensional Convolutional Neural Network used as pre-trained network.(van Stigt, 2020)

van Stigt MN, Ruiz Camps, C, Coutinho, JM, Marquering, H, Doelkahar, BS and Potters, WV. The Effect of Artifact Rejection on the Performance of a Convolutional Neural Network Based Algorithm for Abnormal Versus Normal EEG Data Classification. SSRN (preprint server) 2020.
